# Effect of photodynamic therapy on local muscle treatment in a rat muscle injury model: a controlled trial

**DOI:** 10.1186/s13018-015-0193-9

**Published:** 2015-04-18

**Authors:** Kazuhide Inage, Yoshihiro Sakuma, Kazuyo Yamauchi, Akiko Suganami, Sumihisa Orita, Go Kubota, Yasuhiro Oikawa, Takeshi Sainoh, Jun Sato, Kazuki Fujimoto, Yasuhiro Shiga, Kazuhisa Takahashi, Seiji Ohtori, Yutaka Tamura

**Affiliations:** Department of Orthopaedic Surgery, Graduate School of Medicine, Chiba University, 1-8-1 Inohana Chuo-ku, Chiba City, Chiba 260-8670 Japan; Department of Orthopaedic Surgery, National Hospital Organization Chiba Medical Center, 4 Chome-1-2 Tsubakimori, Chiba City, Chiba Japan; Department of Bioinformatics, Graduate School of Medicine, Chiba University, 1-8-1 Inohana Chuo-ku, Chiba City, Chiba Japan; Department of Orthopaedic Surgery, Teikyo University Chiba Medical Center, 3426-3 Anesaki, Chiba City, Chiba Japan

**Keywords:** Muscle, Injury, Photodynamic therapy

## Abstract

**Background:**

Muscle injury is common and is thought to account for 10%–50% of all sports-related injuries. The use of rest, ice, compression, and elevation is common in clinical practice, but many treatments over a long period are required to produce a therapeutic effect. We evaluated the utility of photodynamic therapy as a new treatment option for the acute stage of muscle injury.

**Methods:**

Twenty 8-week-old Sprague-Dawley male rats underwent experimental injury of the right gastrocnemius muscle with a drop-mass method. After muscle injury was induced, a liposomally formulated indocyanine green derivative (7 mg/kg) near-infrared laser irradiation was performed at 18 h after injury. Local time-dependent changes in the treatment (*n* = 14) and no treatment (*n* = 14) groups were evaluated with *in vivo* imaging, histologic examination, and enzyme-linked immunosorbent assay methods.

**Results:**

*In vivo* imaging fluorescence values were significantly higher in the no treatment group, whereas interleukin-6 and tumor necrosis factor-α levels were significantly higher in the treatment group at 18 h after injury. Histologic examination results revealed that the treatment group had less bleeding and more degeneration repair processes than the no treatment group at 24 h and 1 week after muscle injury.

**Conclusions:**

These findings suggest that photodynamic therapy promotes a tissue-repairing effect during the early stage of muscle injury.

## Introduction

Muscle injuries are common and reportedly account for 10%–50% of all sports-related injuries [1]. These injuries occur when the muscle fibers are damaged by external pressure, often during contact sports such as football, rugby, and American football. Muscle injuries most commonly occur on the frontal surface of the thigh.

The so-called rest, ice, compression, and elevation (RICE) treatment is the most popular acute-phase treatment in clinical practice. However, previous magnetic resonance imaging (MRI)-based studies have shown that a large number of treatments over a long duration are required to produce a therapeutic effect [2]. While echo assessments have shown that the pressure in a local hematoma is controlled, the appropriate treatment duration is unknown, and the acute period of treatment has not been sufficiently established [3]. Importantly, inappropriate treatment in the early phase of muscle injury results in chronic pain in approximately 30% of patients [4]. Therefore, appropriate treatment in the early phase is considered critical for the prevention of the transition to chronic pain. However, the treatment efficacy may depend on the severity of the injury.

**Figure 1:**
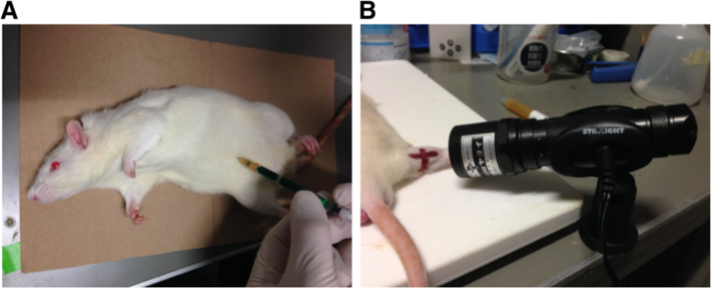
Photodynamic therapy. **(A)** Injecting a light-sensitive substance (photosensitizer) into a biologic body. **(B)** Irradiating the target body tissue with light at a particular wavelength range and evolving active oxygen from the light-sensitive substances.

Recent muscle pain assessments have revealed important roles for cytokines during the transition from the acute to the chronic pain phase. Photodynamic therapy is an operative method of locally treating tumors and inflammation, which involves injecting a light-sensitive substance (photosensitizer) into a biologic body, irradiating the target body tissue with light at a particular wavelength and evolving active oxygen from light-sensitive substances (Figure 1). In particular, near-infrared fluorescent dye-bounded lipid, which tends to readily accumulate in the fragile vasculature of tissues affected by tumors and inflammation, has attracted attention as an effective local treatment option [2,3,5-13]. Indeed, a Japanese study has reported the effectiveness of this treatment for cancer [7]; Japanese National Health Insurance covers the treatment for early cancers of the esophagus, stomach, and other areas. However, while many studies have reported the use of this procedure for tumor therapy, reports on its use for inflammation, as in the case of muscle injury, are lacking. Therefore, the purpose of the present study was to evaluate the use of photodynamic therapy as a new treatment option for inflammation caused by muscle injury.

## Methods

**Figure 2:**
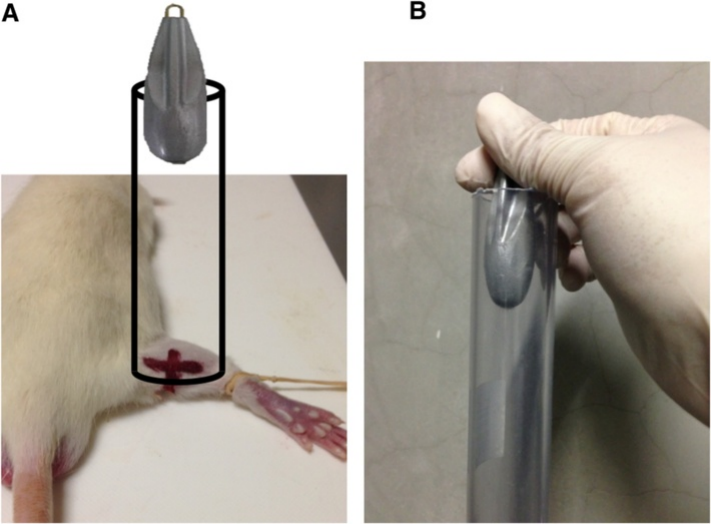
Drop-mass method. **(A)** Schematic representation of the impact apparatus. **(B)** A 115-g weight was dropped from a height of 1 m through an acrylic guide tube. The weight was dropped onto an impacter, dividing the muscle belly without injuring the overlying skin.

**Figure 3:**
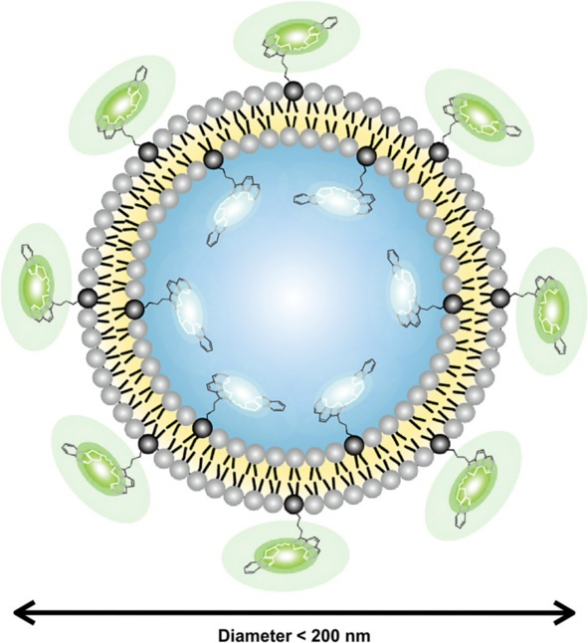
Schematic structure of the liposomally formulated indocyanine green derivative, designated as LP-iDOPE.

**Figure 4:**
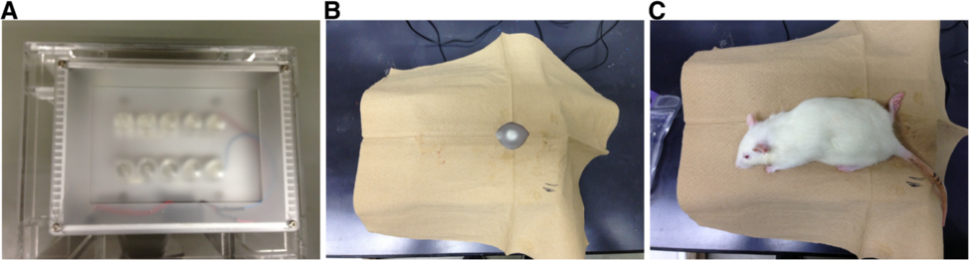
Near-infrared laser irradiation system (light-emitting diode (LED) device, 810 nm; power, 0.1 W/cm^2^; irradiation time, 30 s). **(A)** LED device. **(B)** Covering the LED except the targeting spot. **(C)** Irradiation to the rat at 18 h after muscle injury.

All animal procedures and protocols were approved by the ethics committees of Chiba University, and followed the National Institutes of Health Guidelines for the Care and Use of Laboratory Animals (1996 revision). We used 28 8-week-old Sprague-Dawley male rats. The body weight of each rat was approximately 250 g at the time of muscle injury. Before inducing the injury, all of the rats were anesthetized with ethyl ether. If a withdrawal reflex occurred, additional anesthesia was administered until no response was noted. The muscle injuries were induced without making a skin incision. Experimental injury of the right gastrocnemius muscle was achieved by a drop-mass method [14]. A 115-g mass was dropped from a height of 1 m onto the medial gastrocnemius muscle of the right leg (Figure 2). The gastrocnemius muscle of the left leg was used as the control site. A liposomally formulated indocyanine green derivative (LP-iDOPE, 7 mg/kg, Figure 3) was injected intraperitoneally after the muscle injury was induced. Eighteen hours after injury, near-infrared laser irradiation (light-emitting diode (LED) device, 810 nm; power, 0.1 W/cm^2^; irradiation time, 30 s; Figure 4) was used to activate the photosensitive dye within the right gastrocnemius. As detailed below, we compared the local time-dependent changes between the treatment (*n* = 14) and no treatment (*n* = 14) groups using three methods described below.

### Experiment 1: *in vivo* imaging

**Figure 5:**
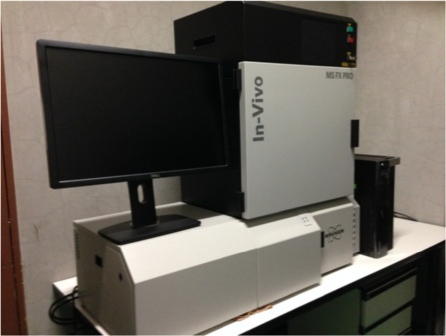
*In vivo* imaging system (In-Vivo FX PRO; BRUKER Corporation, Billerica, MA, USA).

We used an *in vivo* imaging system (In-Vivo FX PRO; BRUKER Corporation, Billerica, MA, USA) to visually evaluate fluorescence values in the two groups at the muscle injury site (right side) at 12, 18, and 24 h after injury (Figure 5). The near-infrared fluorescence *in vivo* imaging system was equipped with an LED that emitted light at a near-infrared wavelength of 760 nm, a charge-coupled device (CCD) as an image detector, and an optical high-pass filter placed in front of the CCD to efficiently detect fluorescence signals. In addition, this system consisted of a camera unit, a controller that operated the camera unit, and a remote controller that controlled the LED intensity, video gain, and offset. The fluorescence image was sent to a digital video processor to be displayed on a television monitor in real time.

### Experiment 2: enzyme-linked immunosorbent assay

Inflammatory mediators in the two groups were analyzed at 18 h after muscle injury. Resected gastrocnemius muscle samples were frozen in liquid nitrogen, pulverized or homogenized, and digested in a tissue lysis reagent. Tumor necrosis factor (TNF)-α and interleukin (IL)-6 production at the muscle injury site were quantified using an enzyme-linked immunosorbent assay in accordance with the manufacturer’s protocols (R&D Systems, Minneapolis, MN, USA). Tissue protein levels were assayed using a kit in accordance with the manufacturer’s protocols (Bio-Rad, Hercules, CA, USA), and inflammatory mediator levels were normalized to tissue protein levels.

### Experiment 3: histologic examination

The muscle injury sites (right side) of the two groups were dissected from the hind limbs under anesthesia with sodium pentobarbital (40 mg/kg, intraperitoneally) at 12, 18, 24 h and 1 week (nine rats; three rats per time period) after the muscle injury and perfused transcardially with 0.9% saline followed by 500 mL of 4% paraformaldehyde in phosphate buffer (0.1 M, pH 7.4). Each formalin-fixed tissue specimen was embedded in paraffin after dehydration for 14 h using an ascending series of ethanol in Tissue-Tek VIP (M1500; Sakura Finetek Japan Co. Ltd., Tokyo, Japan). Sections with a thickness of 4 μm were prepared from these paraffin blocks using a sliding microtome (LS113; Yamato-Kohki Industrial Co. Ltd., Saitama, Japan) and were stuck on a glass slide (#5116; Muto Pure Chemicals Co. Ltd., Tokyo, Japan). These sections were stained with Mayer’s hematoxylin (Muto Pure Chemicals Co. Ltd.) for 5 min after deparaffinization with xylene and ethanol. After washing with distilled water, these sections were dipped in 0.1% ammonium solution several times and washed again, and then washed using 100% ethanol. They were then stained with 1% eosin & phloxine-equivalent solution for 20 s. Finally, the sections were covered with mounting medium (Entellan- New; Merck KGaA, Darmstadt, Germany) after dehydration with a series of ethanol and xylene. The slides were observed under a microscope (BH20; Olympus Corporation, Tokyo, Japan) by a professional animal pathologist, and the degree of each finding was evaluated semiquantitatively. We evaluated the presence or absence of histologic degeneration, bleeding, and neutrophil recruitment in each slice. We also compared the histologic changes between the treatment and no treatment groups.

### Statistical analysis

Fluorescence values at the muscle injury site were compared between the two groups at 12, 18, and 24 h after muscle injury using Student’s *t* test. Levels of inflammatory mediators at the muscle injury site were compared between the two groups at 18 h after the muscle injury using the Student’s *t* test. *P* values of <0.05 were considered statistically significant.

## Results

### *In vivo* imaging

**Figure 6:**
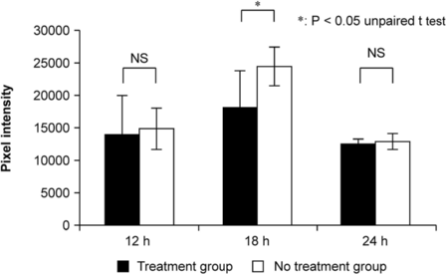
Average fluorescence values in the treatment and no treatment groups using the *in vivo* imaging system.

The average *in vivo* imaging fluorescence values at the muscle injury site of the treatment group were 14,128, 18,193, and 12,429 pixel intensity at 12, 18, and 24 h after injury, respectively. The average *in vivo* imaging fluorescence values at the muscle injury site of the no treatment group were 14,847, 24,375, and 12,834 pixel intensity at 12, 18, and 24 h after injury, respectively. There were no significant differences between the 12- and 24-h fluorescence values in either group (12 h, *P* = 0.780; 24 h, *P* = 0.416). However, the fluorescence value at 18 h after injury in the no treatment group was significantly higher than that in the treatment group (*P* = 0.020, Figure 6). These results indicate that 18 h after injury, and immediately after photodynamic therapy, the fluorescent brightness was significantly decreased in the treatment group compared to that in the no treatment group, suggesting the onset of the a therapeutic effect.

### Enzyme-linked immunosorbent assay

**Figure 7:**
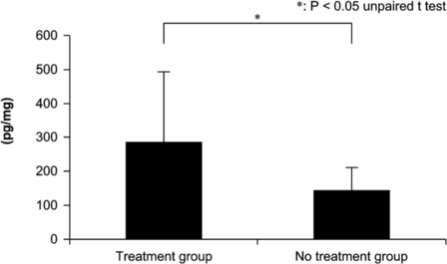
Interleukin-6 levels in the muscle injury site at 18 h after muscle injury in the treatment and no treatment groups.

**Figure 8:**
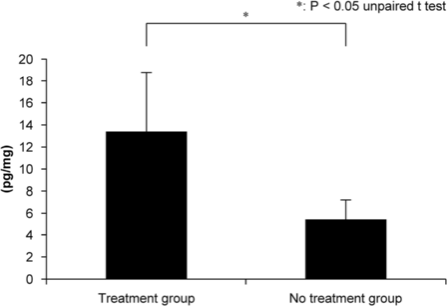
Tumor necrosis factor-α levels in the muscle injury site at 18 h after muscle injury in the treatment and no treatment groups.

IL-6 (Figure 7) and TNF-α (Figure 8) levels were significantly higher in the treatment group at 18 h after muscle injury than in the no treatment group (TNF-α, *P* = 0.001; IL-6, *P* = 0.002). In other words, irritable cytokines were significantly increased in the treatment group compared to the no treatment group at 18 h after muscle injury and immediately after intervention.

### Histologic evaluation

**Figure 9:**
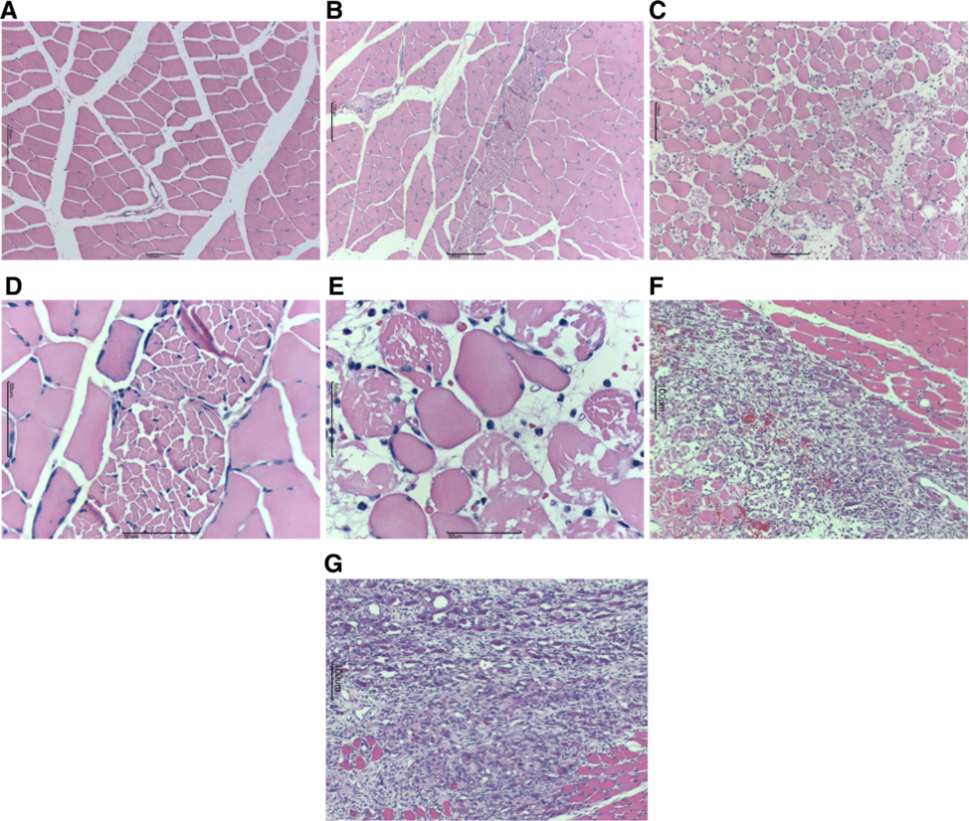
Hematoxylin and eosin staining of local tissues. Transverse sections showing gastrocnemius muscular fibers. **(A)** No injury site (100×). **(B)** Treatment site at 24 h (100×). **(C)**No treatment site at 24 h (100×). **(D)** Treatment site at 24 h (400×). **(E)** No treatment site at 24 h (400×). **(F)** No injury site (100×). **(G)** Treatment site at 1 week (100×).

Local evaluation after staining of the samples with hematoxylin & eosin at 24 h after injury (Figure 9) indicated that the degeneration of tissue was higher in the no treatment group than in the treatment group. Regarding hematoma, the treatment group had almost no bleeding between the muscle bundles, and only a small amount of bleeding in the large breakage hiatus, while the no treatment group showed a greater amount of bleeding. In addition, sparse aggregates of neutrophils were found in the treatment group, whereas the aggregates were dense in the crush-wounded muscle fibers, along with fibroblasts, in the no treatment group. Granulation was strongly recognized in the treatment group compared to that in the non-treatment group during the first week after muscle strain.

## Discussion

In this study, we used *in vivo* fluorescence imaging to evaluate local fluorescent brightness after muscle injury and found that the fluorescence signal was significantly decreased in the treatment group compared to that in the no treatment group at 18 h after injury. According to a previous report, photodynamic therapy has a local hemostatic effect, such as neovascular vessel regression [15]. Indeed, irradiation of LP-iDOPE causes the transformation of local triplet oxygen to singlet oxygen and free radicals; local cellular and tissue damage then occurs, and the local cells that line the blood vessels become damaged and block the blood vessel by creating a neovascular vessel [16]. In actual clinical practice, this mechanism of action is widely used in the ophthalmologic field for age-associated macular degeneration [6]. Our *in vivo* fluorescent findings are in line with this proposed mechanism of action.

The levels of two key cytotoxins reflect the inflammation and repair processes in muscle injury [9-11,17,18]. TNF-α is known not only as an irritable cytokine but also as an apoptosis-inducing factor (repair-starting signal) [8]. In addition, previous reports have indicated that IL-6 plays a central role in inflammation and is involved in the repair and growth of the deep muscle [12]. Thus, increases in irritable cytokine levels are important in the repair of injured tissue. We found that the levels of irritable cytokines significantly increased in the treatment group compared to those in the no treatment group at 18 h after injury and immediately after photodynamic therapy.

 Additionally, histologic evaluations at 24 h after injury demonstrated low bleeding and enhanced degeneration repair processes in the treatment group. In addition, the repair reaction for degeneration tended to be very active even during the first week after muscle strain.

 One limitation of this study is that we did not conduct objective tissue evaluation (i.e., grading for damage). In addition, we did not assess the maximum depth reached by the treatment method. Since these two criteria can be important for the clinical application of photodynamic therapy, we plan to examine them further in the future.

 The LP-iDOPE used in this study was a liposomal preparation including ICG as a fluorescent dye. Although no reports have been published on the clinical use of this agent, the side effects are considered to be the same as those for ICG. ICG is a relatively safe trial drug with few side effects, but side effects (36/21,278 administration cases) were reported in 0.17% of cases in previous studies. The primary side effects reported were shock symptoms (0.02%: five cases), nausea/vomiting (0.08%: 16 cases), angialgia (0.04%: eight cases), and fever/heat sensations (0.02%: four cases) [19-21].

## Conclusions

In conclusion, these findings suggest that photodynamic therapy promotes cytokine-mediated processes. We therefore suggest that photodynamic therapy elicits a tissue-repairing effect during the early stage of injury. Overall, these findings suggest that photodynamic therapy is a beneficial new treatment method for the acute period after muscle injury.
